# Flexural strengthening of RC beams using basalt textile reinforced mortar: experimental and analytical investigation

**DOI:** 10.1038/s41598-026-37322-3

**Published:** 2026-02-19

**Authors:** Ayman Shamseldein, Fareed ELgabbas, Mohamed Kohail, Hany Elshafie

**Affiliations:** Structural Engineering Department, Faculty of Engineering, Ain Shams University, Cairo, Egypt

**Keywords:** Textile-reinforced mortar, Flexure behavior, Reinforced concrete beams, Basalt fiber, Mechanical anchorage, Engineering, Materials science

## Abstract

This study investigates the flexural behavior of RC beams strengthened with Basalt Textile Reinforced Mortar (BTRM), focusing on the influence of the number of textile layers, mesh size, and anchorage techniques. Six full-scale RC beams were tested under four-point bending, comprising one unstrengthened control specimen and five beams strengthened using different BTRM configurations. The experimental results demonstrated that increasing the number of BTRM layers from three to five enhanced the ultimate load capacity by up to 18% compared to the control beam. Nevertheless, debonding was identified as the predominant failure mode across most strengthened specimens. The influence of mesh size was examined by comparing an eight-layer specimen using 5 mm mesh size with a three-layer specimen using 34 mm mesh size; both configurations exhibited comparable flexural performance. Variations in mesh size (34 versus 5 mm) had a negligible effect on load capacity. The incorporation of basalt bars resulted in a marginal improvement in flexural strength, whereas mechanical anchorage provided limited enhancement in overall performance. These findings highlight the critical need to improve bond behavior and anchorage efficiency in order to fully benefit from the strengthening potential of BTRM systems. In addition, an analytical study was conducted to assess the accuracy of existing predictive models, including those proposed in current design guidelines and previously published analytical approaches, against the experimental results. A modified predictive equation derived from an existing analytical model demonstrated good agreement with both the experimental data and results reported in the literature.

## Introduction

The flexural capacity is the most crucial factor to consider when assessing the serviceability of reinforced concrete (RC) beams in actual structures. The need for external strengthening is becoming more prominent due to issues such as corrosion of internal reinforcement, deterioration of concrete strength from aging or environmental exposure, and increased load demands^1^. Fiber Reinforced Polymer (FRP) has become widely adopted as a structural material in many sectors because of its significant benefits^2–6^. Although using FRP was started in the 1970s, its usage has grown substantially in recent years, driven by extensive research aimed at improving structural performance^7^. FRP is a composite material made by combining high-strength fibers with an organic polymer matrix, resulting in a versatile and durable product. Research has shown the advantages of FRP, especially its high strength-to-weight ratio, which facilitates quicker and more efficient installation. Despite these advantages, FRP also has limitations, including significant strength reduction at temperatures around 80°C and challenges in applying it to wet surfaces. In addition to traditional fiber-reinforced polymer (FRP) strengthening systems, other composite-based techniques such as Steel-Reinforced Polymer (SRP) have been successfully used for flexural and shear strengthening of reinforced concrete members^8^. These techniques share similarities in application and failure modes with FRP systems but involve metallic reinforcement embedded in a polymer matrix, offering alternative mechanical and durability characteristics. Experimental studies on SRP-strengthened RC beams have reported improvements in flexural capacity and ductility, with failure mechanisms often governed by interface debonding similar to FRP systems^8,9^.To address the drawbacks of epoxy in FRP and SRP systems, researchers have explored the use of cement-based mortars, which provide improved resistance to high temperatures and can be applied to damp surfaces. Nevertheless, a common challenge with these mortars is the weak bond formed between the fiber sheets and the mortar, often resulting in early failure. To enhance this bond, scientists modified the fiber sheets by incorporating openings of various sizes, which greatly improved adhesion. This advancement led to the development of a new system called Textile Reinforced Mortar (TRM), which integrates open fiber sheets with cement-based mortar. TRM retains the benefits of traditional FRP systems while overcoming their primary limitations^3^ .

Numerous studies have explored the flexural strengthening of reinforced concrete (RC) beams using Textile Reinforced Mortar (TRM), focusing on different materials, configurations, and techniques to enhance performance. Triantafillou and Papanicolaou^10^ investigated the flexural strengthening of RC beams using TRM reinforced with high-strength carbon fiber and compared it to Fiber Reinforced Polymer (FRP) systems. Their findings showed that FRP outperformed TRM by 30% in flexural capacity, specimens strengthened with FRP failed due to fiber rupture which is a preferred failure mode while specimens strengthened with TRM system failed by debonding. D'Ambrisi and Focacci^2^ examined the effects of varying the number of TRM layers (1 to 4) and textile types (carbon and Poly(p-phenylene-2,6-benzobisoxazole (PBO)) on beams’ performance. They found that flexural capacity increased by increasing the number of layers and that mortar type significantly influenced TRM effectiveness. Failure modes included fiber sliding and premature TRM debonding at the concrete-mortar interface. Ombres^11^ studied PBO-TRM strengthened beams, focusing on the number of plies (1 to 3), internal reinforcement ratios, and TRM anchorage length. Results showed that TRM enhanced flexural strength by 10% to 44%, depending on reinforcement and ply count. Failure modes varied from fiber sliding in single-ply specimens to debonding in multi-ply beams, with insufficient TRM bonding lengths leading to sudden failures. Elsanadedy et al.^12^ evaluated basalt-TRM systems by testing different mortar types and up to 10 layers of basalt textiles. Polymer-modified cement mortars outperformed traditional mortars. The author concluded that using 10 plies of basalt textile reinforced mortar (BTRM) increased flexural capacity by 90%. While TRM was less effective than FRP in strength enhancement, it offered superior deformation enhancement. Yin et al.^13^ explored the effect of TRM layer count (1 to 3) and textile surface treatments on flexural performance. Carbon fibers were used in the loading direction and glass fibers transversely, with sand coatings applied to textiles. Strength gains ranged from 10 to 45%, while surface treatments had a minimal impact on performance. Babaeidarabad et al.^14^ analyzed the influence of plies number (1 to 4) and concrete compressive strength on flexural capacity. Beams with lower concrete strength showed increases ranging from 32 to 92% in flexural capacity, while beams with higher strength exhibited flexural capacity improvements ranging from 13 to 73%, indicating that substrate properties significantly affect TRM effectiveness. Ebead et al.^15^ investigated the effects of internal reinforcement ratios, textile types (Carbon and PBO), and plies count (1 to 3) on flexural strength. Carbon-TRM beams achieved strength increases between 14 and 77%, while PBO-TRM beams saw gains of 8% to 27%. Failure modes varied based on ply count, with fiber slippage in lower-ply specimens and debonding in beams with more plies. Basma et al.^16^ studied two-span of RC beams strengthened with PBO-TRM in both hogging and sagging regions. They found that the hogging-to-sagging strengthening ratio influenced failure modes in hogging regions but had no impact on sagging sections. Load-carrying capacity was primarily affected by the strengthening configuration and the number of TRM layers. Shamseldein et al .^4^ published a review article on strengthening RC beams using TRM. The review summarized that the textile-fiber materials, the number of plies, the strengthening configuration, the concrete compressive strength, the type of textile-fiber materials, and the strengthening system (i.e. TRM versus FRP) were all examined parameters in the previously mentioned studies in the area of strengthening RC beams with TRM. The main findings were as follows: (a) applying TRM reinforcement to RC beams led to a notable improvement in their flexural capacity; (b) increasing the number of TRM layers further enhanced flexural strength and altered the failure mode from slippage to rupture. Triantafillou and Papanicolaou, based on tests with two specimens, observed that TRM was approximately 30% less effective than FRP. In contrast, Elsanadedy et al.^12^found that while TRM was only slightly less effective than FRP in enhancing flexural capacity, it was significantly more efficient in improving deformation capacity. This conclusion was reached after testing two specimens, one with five layers of TRM in the form of a U-shaped basalt fibre textile jacket. While the other one was using FRP. Based on the findings of the preceding experiments, it is concluded that more research into the efficiency of basalt in flexural strengthening of RC beams is required. Koutas et al.^17^ studied the effect of mortar type on the efficiency of strengthening RC beams using basalt and glass textiles with different mesh sizes. It was concluded that the type of mortar has a high effect on flexure performance in case of debonding failure. Also, it was found that using bigger mesh sizes is more preferable than small mesh sizes. Holsamudrkar et al.^18^ studied the effect of end anchorage on the capacity of RC beams. It was concluded that the anchorage-based approach enhances the performance by approximately 16%. Additionally, the failure mode shifts from fiber–fiber slip to a combination of fiber rupture and fiber-matrix slip. Holsamudrkar et al.^19^ also investigated the impact of applying mechanical anchorage along the entire span of the beam. The use of mechanical anchorage resulted in a 21% increase in load-carrying capacity compared to conventionally strengthened beams. Moreover, the failure mode shifted from fabric-matrix debonding to fabric rupture. Holsamudrkar et al.^18^studied the effect of impregnation and anchorage on beams strengthened with TRM. Their findings indicated that the ultimate strain of members strengthened with TRM and anchorage could be safely increased from 0.012 to 0.016. This highlights the crucial role of anchorage in enhancing the effectiveness of TRM systems. Recent advances in data-driven modeling have enabled prediction of full load–deflection behavior of FRCM-strengthened beams. For example, a study by Daneshvar et al.^20^developed machine learning models that predict key points on the load–deflection curve of FRCM-strengthened beams by considering geometric and mechanical properties of the beam and strengthening system, including number of layers, fabric type, and presence of anchorage. Their models achieved low prediction errors for ultimate load, stiffness, and absorbed energy, indicating the potential of ML techniques for rapid and accurate assessment of global structural response.

Despite the extensive research on textile-reinforced mortar (TRM) systems for flexural strengthening of reinforced concrete (RC) beams, several gaps remain, particularly concerning the use of basalt textiles and the effectiveness of different bond-enhancement strategies. Limited experimental data are available on the influence of basalt textile mesh size, the number of textile layers, the use of embedded basalt bars, and mechanical anchorage on the flexural behavior and failure mechanisms of strengthened RC beams. Moreover, existing analytical models often assume idealized bond conditions, which may not accurately reflect debonding-governed behavior observed in practice.

In this context, the present study aims to experimentally investigate the flexural behavior of RC beams strengthened with basalt textile-reinforced mortar (BTRM). The specific objectives are to evaluate the effects of (i) the number of textile layers, (ii) textile mesh size, (iii) the incorporation of embedded basalt bars, and (iv) the use of mechanical anchorage on load-carrying capacity, stiffness, ductility, energy absorption, and failure modes. Six full-scale RC beams, including one control specimen and five strengthened configurations, were tested under four-point bending.

The novelty of this study lies in providing a systematic experimental assessment of basalt-based TRM systems considering multiple interacting parameters, along with a critical evaluation of existing analytical models. In addition, a modified predictive equation is proposed to improve the estimation of flexural capacity under partial-bond conditions. The outcomes of this research contribute to a more realistic understanding of BTRM strengthening effectiveness and offer practical guidance for the design and optimization of TRM-strengthened RC members.

## Experimental program

### Material properties

**Fig. 1:**
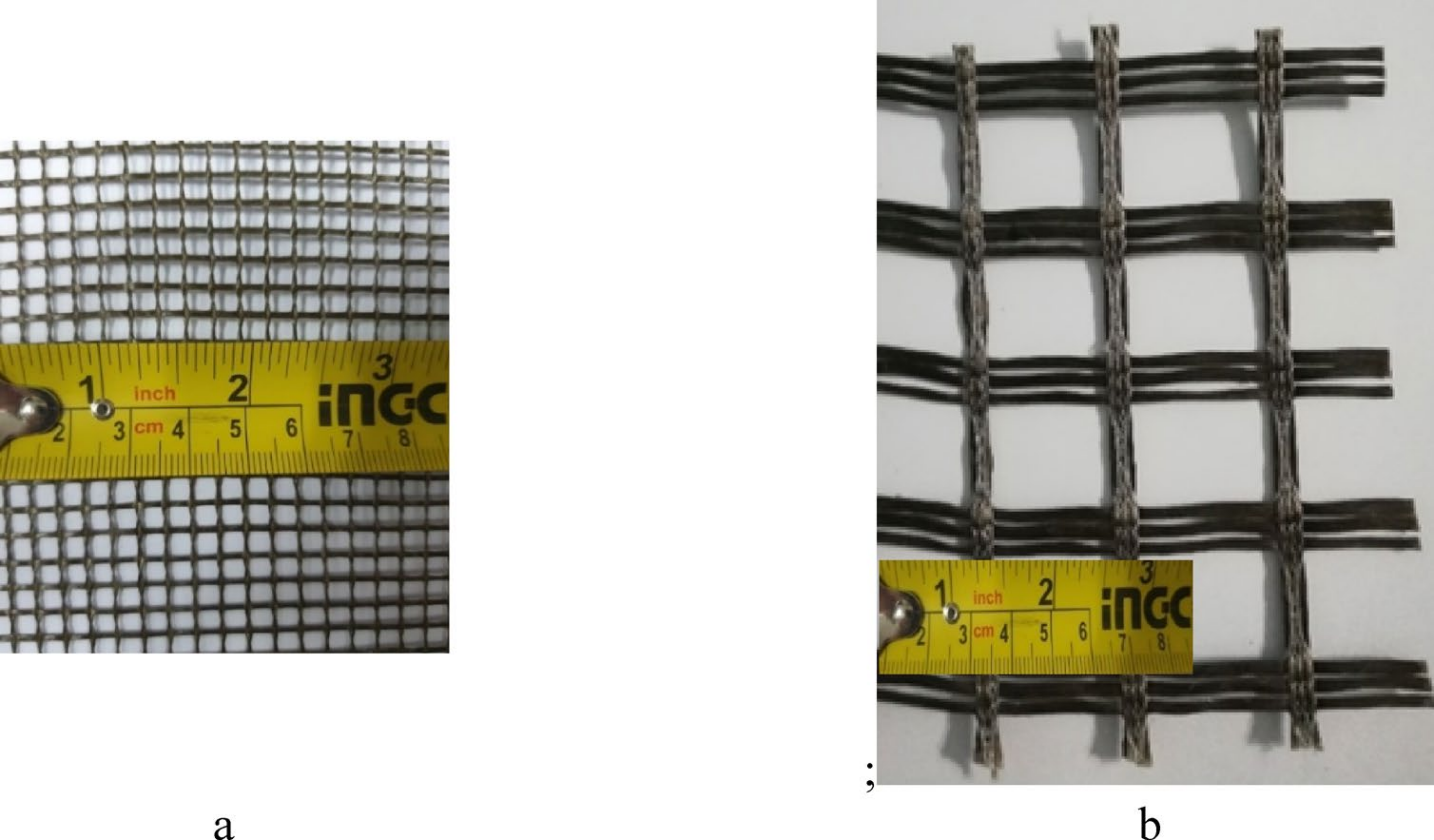
Configurations of the basalt textiles used: (**a**) Mesh with a 5 mm opening (M5), (**b**) Mesh with a 34 mm opening (M34).

**Fig. 2:**
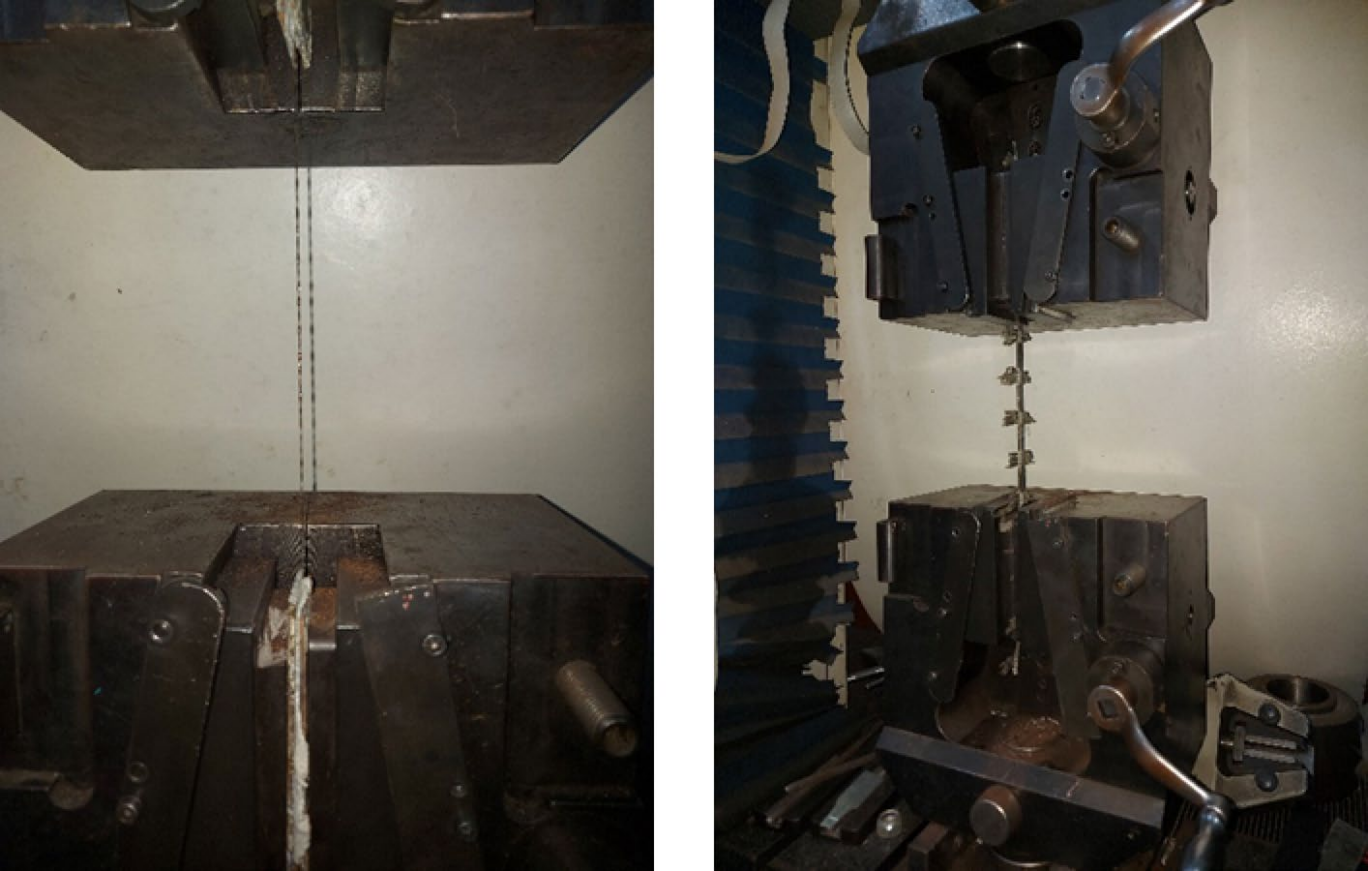
Test setup for textile in tension.

Two types of basalt textiles, featuring opening sizes of 5 mm and 34 mm (designated as M5 and M34, respectively), were used in this study, as presented in Fig. 1. These textiles were produced in China. The weight-equivalent thicknesses of the M5 and M34 textiles were 0.030 mm and 0.077 mm, respectively. Their mechanical properties were determined by testing three tensile specimens, as shown in Fig. 2, in accordance with shamseldein et al.^5^. The modulus of elasticity was calculated using the chord modulus between 30 and 60% of the textile’s ultimate tensile strength, in accordance with Younis et al.^21^. Table 1 provides the mechanical characteristics of the textiles tested.

**Table 1:** The average mechanical properties of basalt textiles.

Textile Mesh Size (mm)	Mesh opening area (mm^2^)	Equivalent textile thickness (mm)	Equivalent textile area per 100 mm length (mm^2^/100mm)	Tensile strength (MPa)	Ultimate strain (%)	Tensile modulus of elasticity (GPa)
5 (1.2%)	25	0.030	3	810 (8.1%)	2.8 (9.5%)	29 (8.7%)
34 (2.9%)	1156	0.077	7.7	980 (8.8%)	2.5 (9.8%)	38 (9.3%)

The coefficient of variation is in parentheses.

The mortar used in the TRM system was a polymer-modified type incorporating short glass fibers, with a compressive strength of 23.5 MPa. Crushed dolomite with a maximum size of 20 mm was used as the coarse aggregate, while natural sand served as the fine aggregate. The bulk specific gravity and unit weight were 2.68 and 1.60 t/m^3^ for the coarse aggregate, and 2.67 and 1.63 t/m^3^ for the fine aggregate, respectively. The compressive strength of the polymer-modified mortar was determined in accordance with EN 1015–11^22^.

Cement type CEM I 42.5R was used, conforming to the EN197 standard^23^. The concrete achieved a compressive strength of 25 MPa after 28 days. The mix proportions are detailed in Table 2. The compressive strength of concrete was determined using standard cube tests in accordance with BS EN 123903^24^.

**Table 2:** Concrete mix proportions (kg/m^3^).

Concrete Class	Cement	Coarse aggregate	Fine aggregate	Water	Superplasticizer
C25	300	1200	600	210	4.5

**Fig. 3:**
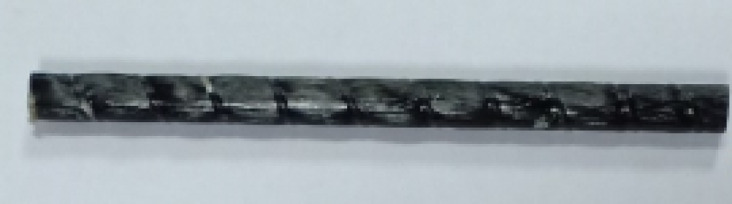
Basalt FRP bars of 6 mm diameter used for specimens.

In addition, two plain basalt bars of diameter 6 mm were used in this investigation, as shown in Fig. 3. The tensile strength of the bars was 1570.3 MPa and the modulus of elasticity was 60.3 GPa, whereas the ultimate strain was 2.6%.The tensile properties of the basalt bars were evaluated following ASTM D7205/D7205M^25^ , which is commonly used for testing fiber-reinforced polymer bars.

The longitudinal steel reinforcement consisted of deformed steel bars with a nominal diameter of 12 mm with yield strength of 400 MPa and tensile strength of 600 MPa. The mechanical properties of the steel reinforcement were determined following BS EN ISO 15,630–1^26^.

### Test specimens

**Fig. 4:**
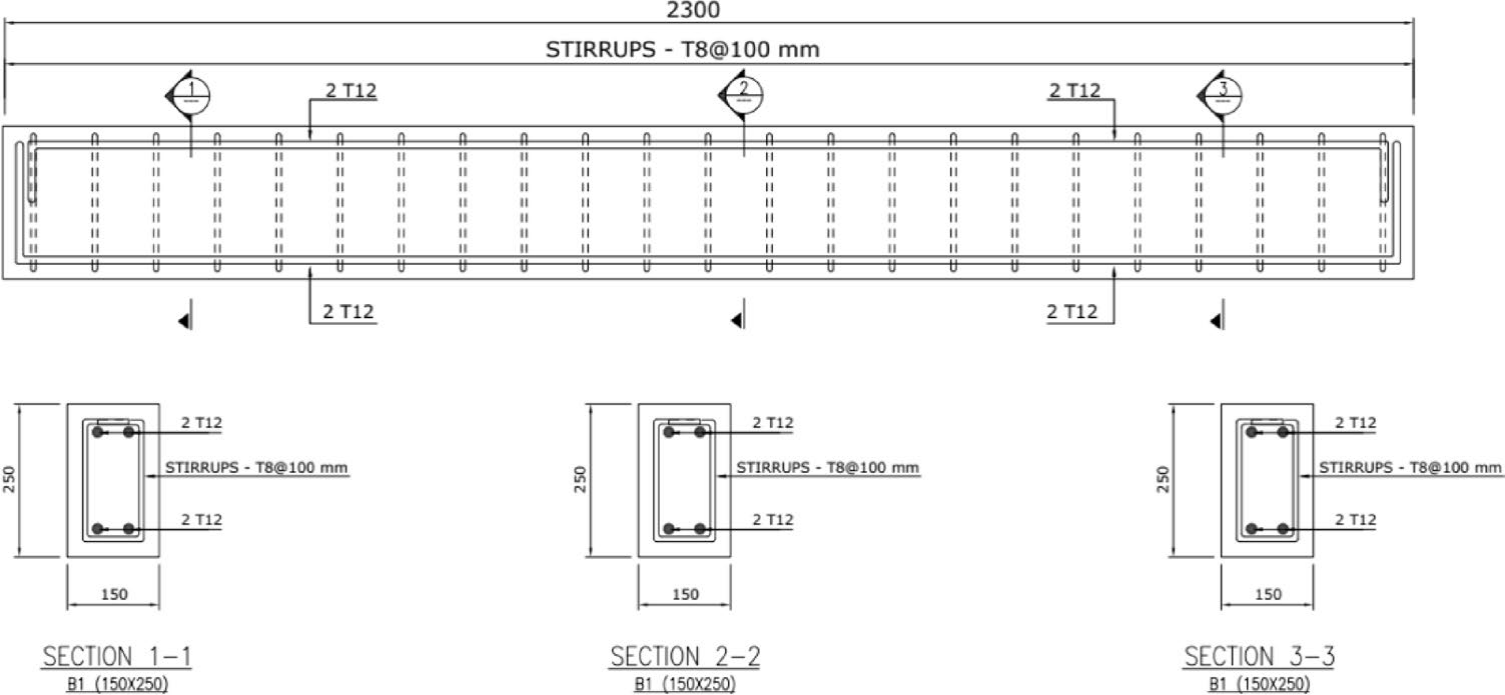
Reinforcement details for specimens.

All concrete beams measured 2.30 m in total length, 150 mm in width, and 250 mm in overall height, as shown in Fig. 4. All beams were reinforced in flexure using two deformed steel bars of 12 mm diameter at the top and bottom. The concrete cover was fixed for all specimens at 25 mm. The bending reinforcement is selected to get an under-reinforced section. This means that failure shall be occurred in yielding of bottom steel reinforcement. Shear reinforcement consisted of stirrups made of plain bars of 8 mm diameter of 100 mm spacing.

The specimens were cast in wooden molds.The interior faces of the molds were oil-coated before installing the reinforcement cage in the molds. The required concrete cover is insured by supporting the cages in the mold with plastic chairs. Reinforcement was placed in the molds before casting. The concrete was compacted using a hand-held vibrator. The top surface of the specimens was leveled and finished using a hand trowel. Specimens were extracted from the molds after 48 h after casting, covered with wet burlap for seven days, and stored under laboratory conditions till the testing day. Six 158 mm cubes were cast from different concrete batches, de-molded the next day after casting, and wet cured till testing date.

For the strengthening procedure, firstly all specimens were roughened using a hammer and chisel to enhance the bond between TRM and concrete. Surface preparation was performed using a hammer and chisel to remove the weak surface laitance and locally expose the coarse aggregate, following common practice in TRM/FRCM strengthening applications. The roughening depth was limited and carefully controlled to avoid inducing microcracking or altering the effective cross-section of the beams. Since the same surface preparation procedure was applied to all strengthened specimens, its effect on the global flexural response is considered uniform and does not influence the comparative assessment of different strengthening configurations.

**Fig. 5:**
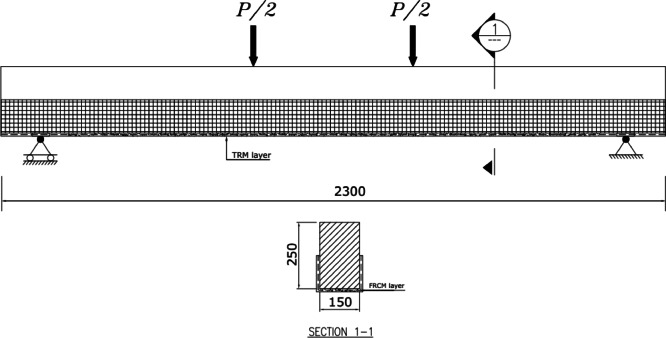
Strengthening scheme for specimens.

The strengthened RC beams were externally strengthened by applying TRM on the bottom face and the sides. The procedure of applying the strengthening system has first applied a layer of mortar then the layer of textile was pressed into the mortar. After that, a layer of mortar was introduced above the textile layer, and this procedure was repeated until all plies were applied. The strengthening scheme is presented in Fig. 5. The BTRM strengthening was applied in a U-shaped configuration, fully covering the bottom face of the beam and extending vertically along both sides for a height of 125 mm, corresponding to the full depth of the beam cross-section. Each mortar layer had an average thickness of approximately 3 mm. Consequently, the total thickness of the BTRM strengthening system depended on the number of textile plies applied, resulting in an overall thickness of about 9 mm, 15 mm, and 24 mm for specimens strengthened with 3, 5, and 8 layers, respectively.

**Fig. 6:**
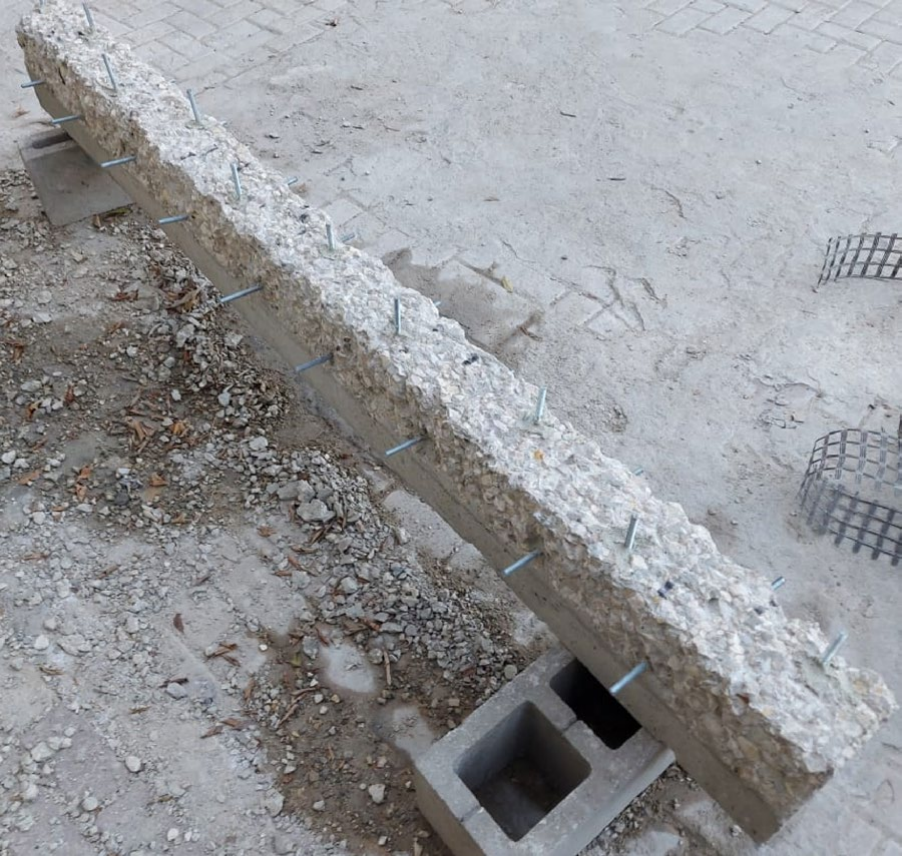
Configuration of mechanical anchors.

For specimens reinforced with mechanical anchors, holes of diameter 14 mm were drilled firstly with 10 mm depth. Then, the holes were cleaned from dust using compressed air. The drilled holes were filled with Sikadur®-31^27^ CF Normal, a two-component, high-strength structural epoxy adhesive manufactured by Sika, and the mechanical anchors were then inserted in accordance with the manufacturer’s installation guidelines The configuration of the mechanical anchors is shown in Fig. 6. The mechanical anchorage system investigated in this study can be practically implemented in real-world strengthening applications using conventional construction techniques and commercially available materials. The anchorage procedure involves drilling shallow holes in the concrete substrate at predefined locations, cleaning the holes to remove dust and debris, and installing anchors bonded with structural epoxy. This process is compatible with standard site practices and does not require specialized equipment beyond common drilling and injection tools.

### Experimental variables and specimen details

The test variables investigated in this study included the textile mesh size, the number of textile plies, the use of mechanical anchorage, and the incorporation of basalt bars. Two plain basalt bars with a diameter of 6 mm were used where applicable. Two textile mesh sizes were considered, namely 5 mm and 34 mm. Each specimen listed in Table 3 was assigned a designation code based on the strengthening configuration. The first part of the code denotes the textile mesh size (M5 or M34), followed by the number of plies (3, 5, or 8). The symbol ‘B’ indicates the inclusion of basalt bars, while ‘A’ denotes the use of mechanical anchorage. For example, specimen M34-5-A represents a beam strengthened with five layers of M34 basalt textile and mechanical anchorage.

**Table 3:** Specimen details.

No	Code	Textile mesh size (mm)	Number of plies	Basalt bars	Mechanical anchorage
01	C	–	–	–	–
02	M34-3	34	3	–	–
03	M34-5	34	5	–	–
04	M34-5-B	34	5	YES	–
05	M34-5-A	34	5	–	YES
06	M5-8	5	8	–	–

### Specimen preparation and testing

**Fig. 7:**
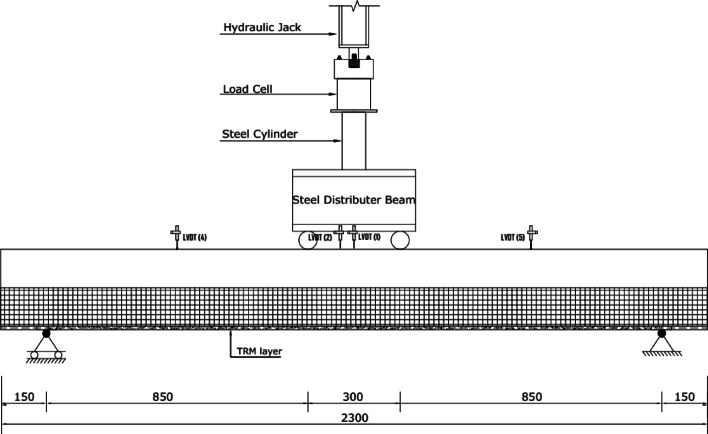
Schematic for test setup.

A schematic of the test setup is shown in Fig. 7. The specimens were tested under four-point load. A hydraulic jack of 300 kN capacity was used to apply the load consistently. A load cell of 450 kN capacity was used to measure the load. The vertical displacements at mid-span and quarter-span were measured using linear variable displacement transducers (LVDTs).

## Test results and discussion

**Fig. 8:**
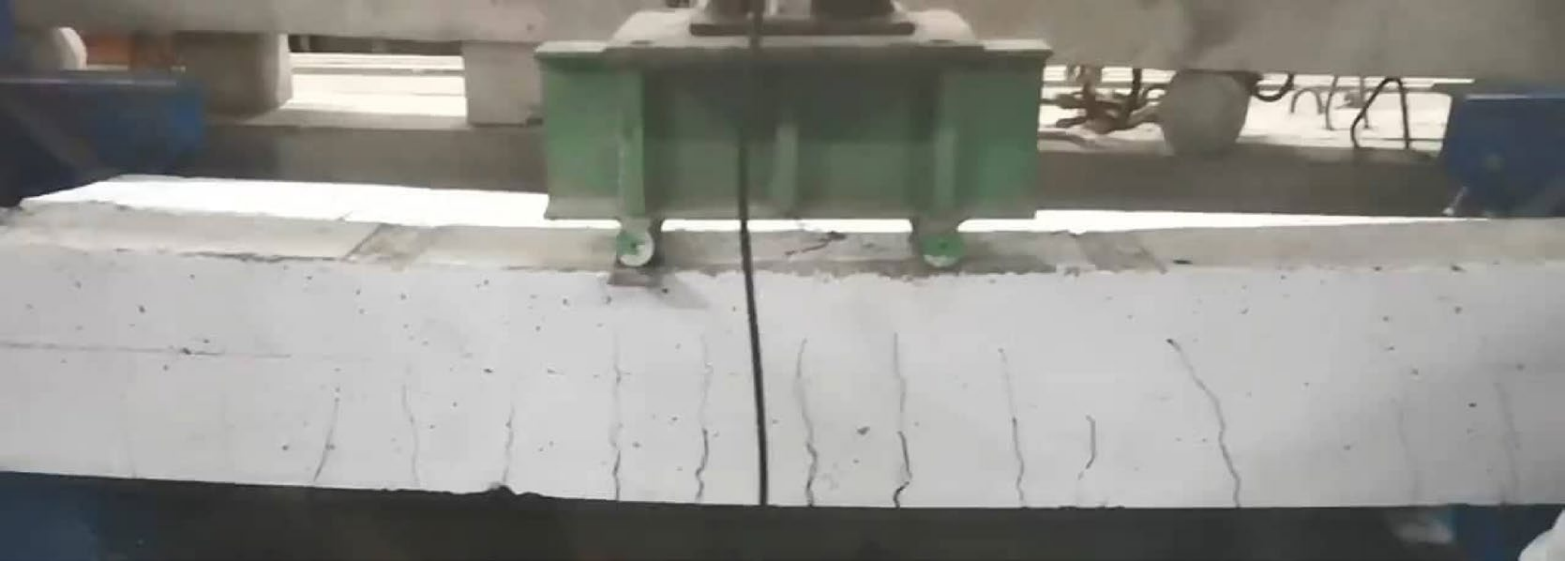
Typical steel yielding failure mode for control specimen.

Table 4 summarizes the test results of the experimental work presented as ultimate load capacity (P_u_) and failure mode as well as the capacity enhancement ratio compared to the un-strengthened beam. Figure 8 shows the load displacement curves for all specimens.

**Table 4:** Results of parameters evaluated in specimens.

Specimen	Ductility Ratio (μ)	cracked Stiffness (tanα) (kN/mm)	Energy Absorption (J)	δ_y_ (mm)	δ_u_ (mm)	P_y_ (kN)	P_u_ (kN)	Enhancement (%)	Failure mode
C	1.8	3.8	2152	10	18	59.5	70.0	–	Steel yielding
M34-5-A	2.7	2.5	2330	11	30	65.9	77.6	11	Debonding
M5-8	2	35	2391	8	16	67.1	81.0	15	Debonding
M34-3	1.5	5.9	2410	8	12	68.8	81.0	15	Debonding
M34-5-B	2.5	3	2548	10	25	70.5	83.0	18	Debonding
M34-5	2.4	3.5	2390	9	22	68.8	81.0	15	Debonding

The ductility ratio, cracked stiffness, and energy absorption of all specimens were evaluated based on the experimental load–deflection response using standard definitions commonly adopted in the literature derived by Akin et al.^28^. The yield displacement (δ_y_) was determined using a bilinear idealization of the experimental load–deflection curve. The initial elastic stiffness was defined from the linear portion of the response up to first yielding of the tensile steel reinforcement. This stiffness was then extended until it intersected a horizontal line corresponding to the yielding load (P_y_), and the corresponding displacement at this intersection was taken as the yield displacement (δ_y_). The ultimate displacement (δ_u_) was defined as the displacement corresponding to the peak load. The cracked stiffness reported in Table 4 was calculated as the ratio between the failure load (P_u_) and the corresponding ultimate displacement (δ_u_). This approach was adopted to provide a simplified global representation of the post-cracking stiffness of the beams, particularly for specimens exhibiting nonlinear response and debonding-controlled failure.

### Failure modes

**Fig. 9:**
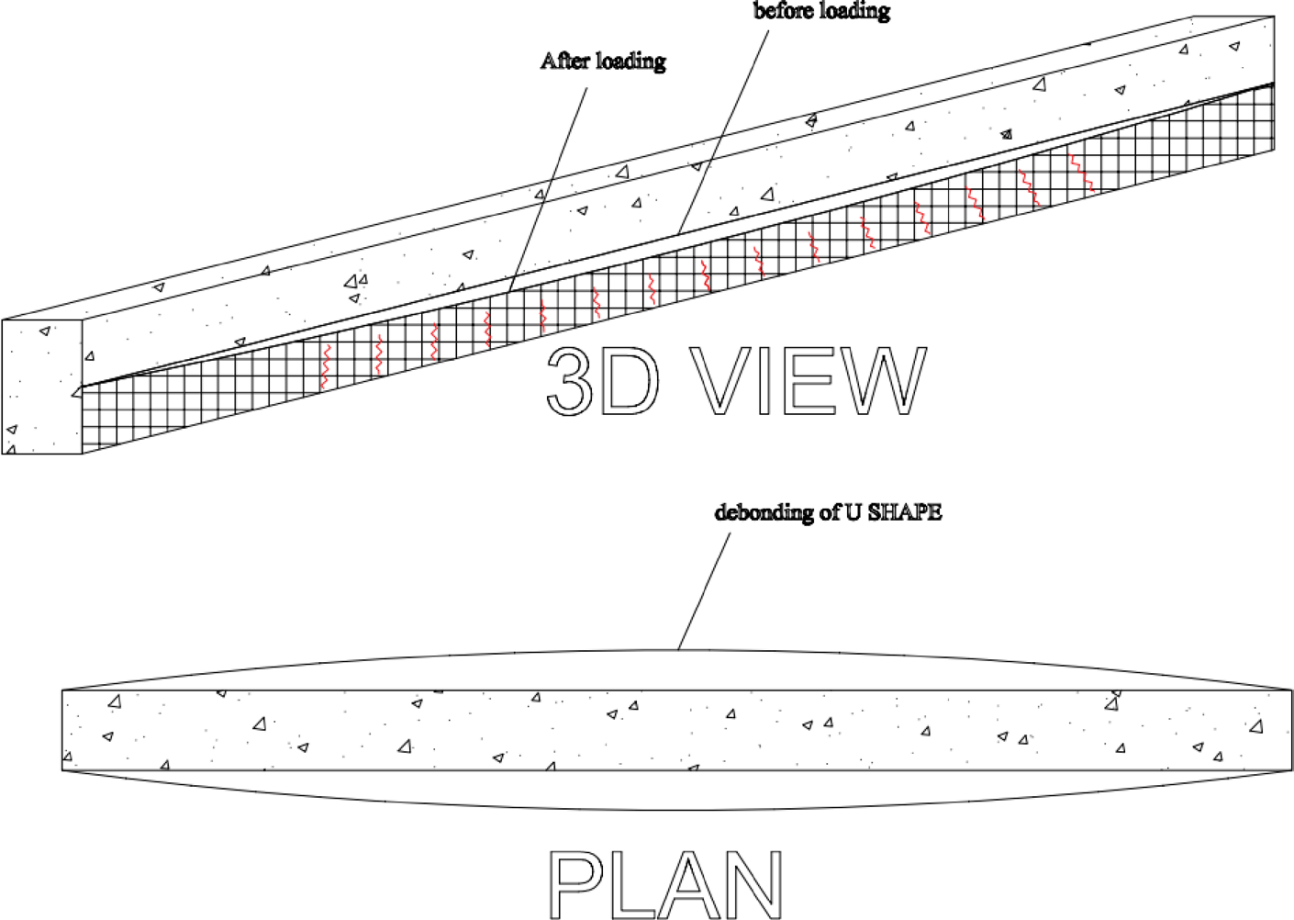
Typical failure mode for all repaired specimens by debonding.

**Fig. 10:**
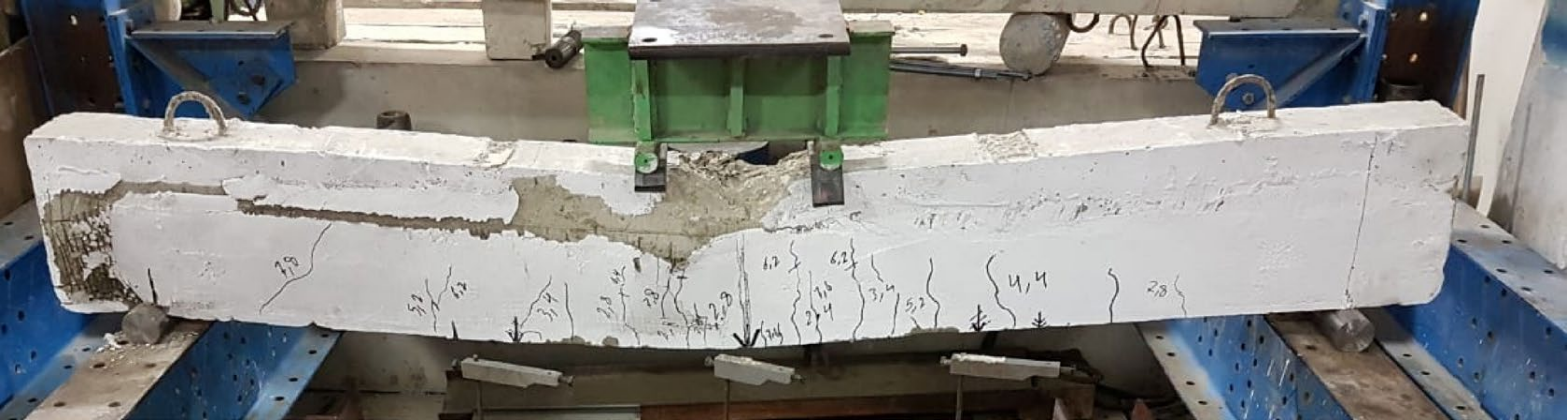
Failure mode of M34-5-B specimen.

**Fig. 11:**
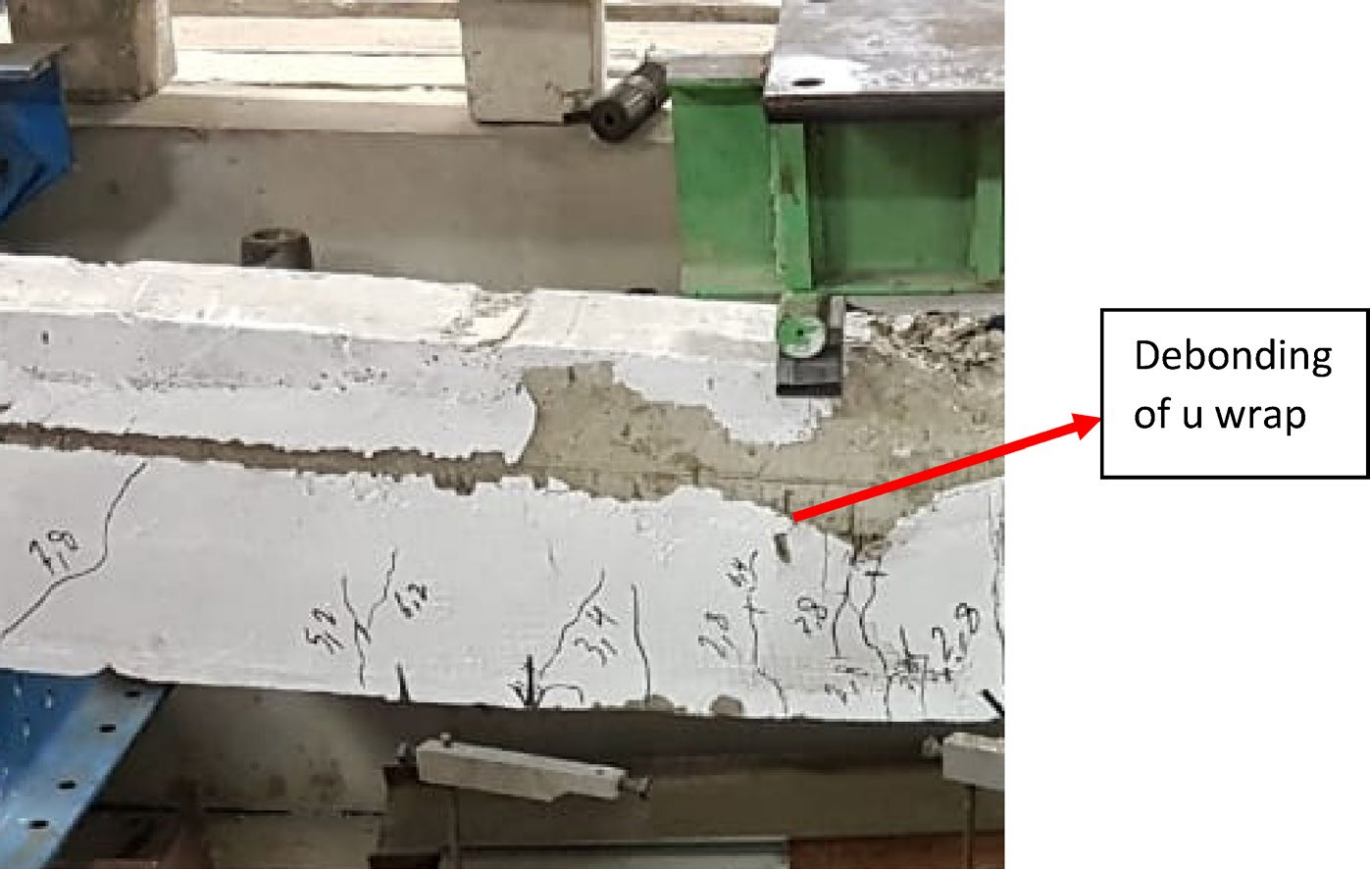
Enlarged photo of the debonding failure mechanism in beam M34-5-B.

**Fig. 12:**
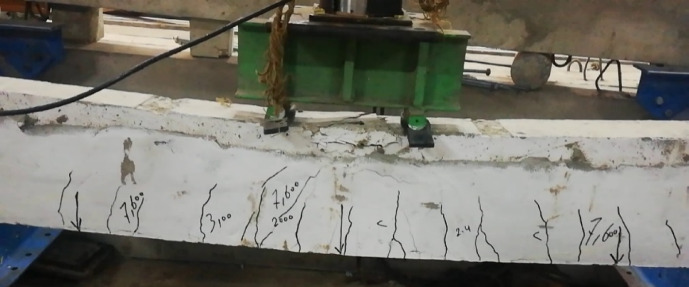
Failure mode for M34-5 specimen.

**Fig. 13:**
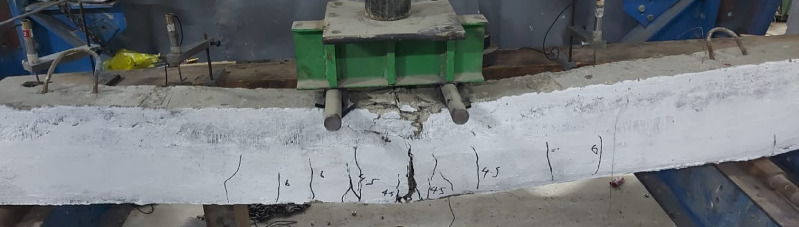
Failure mode for M34-3 specimen.

**Fig. 14:**
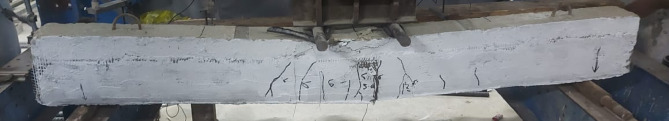
Failure mode for M5-8 specimen.

**Fig. 15:**
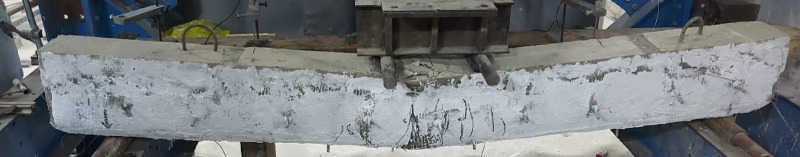
Failure mode for M34-5-A specimen.

Failure mode for all specimens is shown in Figs. 8, 9, 10, 11, 12, 13, 14, 15. The control specimen reflected the typical under-reinforced behavior, since the steel reinforcement reached the yield stress before concrete crushing. Figure 8 shows cracks’ pattern for control specimen. For the repaired specimens, identifying the mode of failure was challenging due to the presence of the strengthening system that covered the crack propagation. However, the failure modes can be obtained from the load capacity of the beams and observations. Since the load capacity of the beams is almost the same, it suggests a dominant failure mode was prevalent across all specimens, preventing any significant increase in capacity. The predicted failure mode is likely debonding. The debonding was notices in the u wrap that confines the TRM strengthening as shown in Fig. 10. Post-test inspection revealed that debonding primarily occurred at the concrete–mortar interface. The BTRM layers detached as an intact composite system, with the mortar and embedded textiles remaining bonded to each other. The exposed concrete surface after failure indicates that debonding was governed by loss of adhesion at the concrete substrate rather than cohesive failure within the mortar layers or slippage at the textile–mortar interface.

### Load deflection curves

**Fig. 16:**
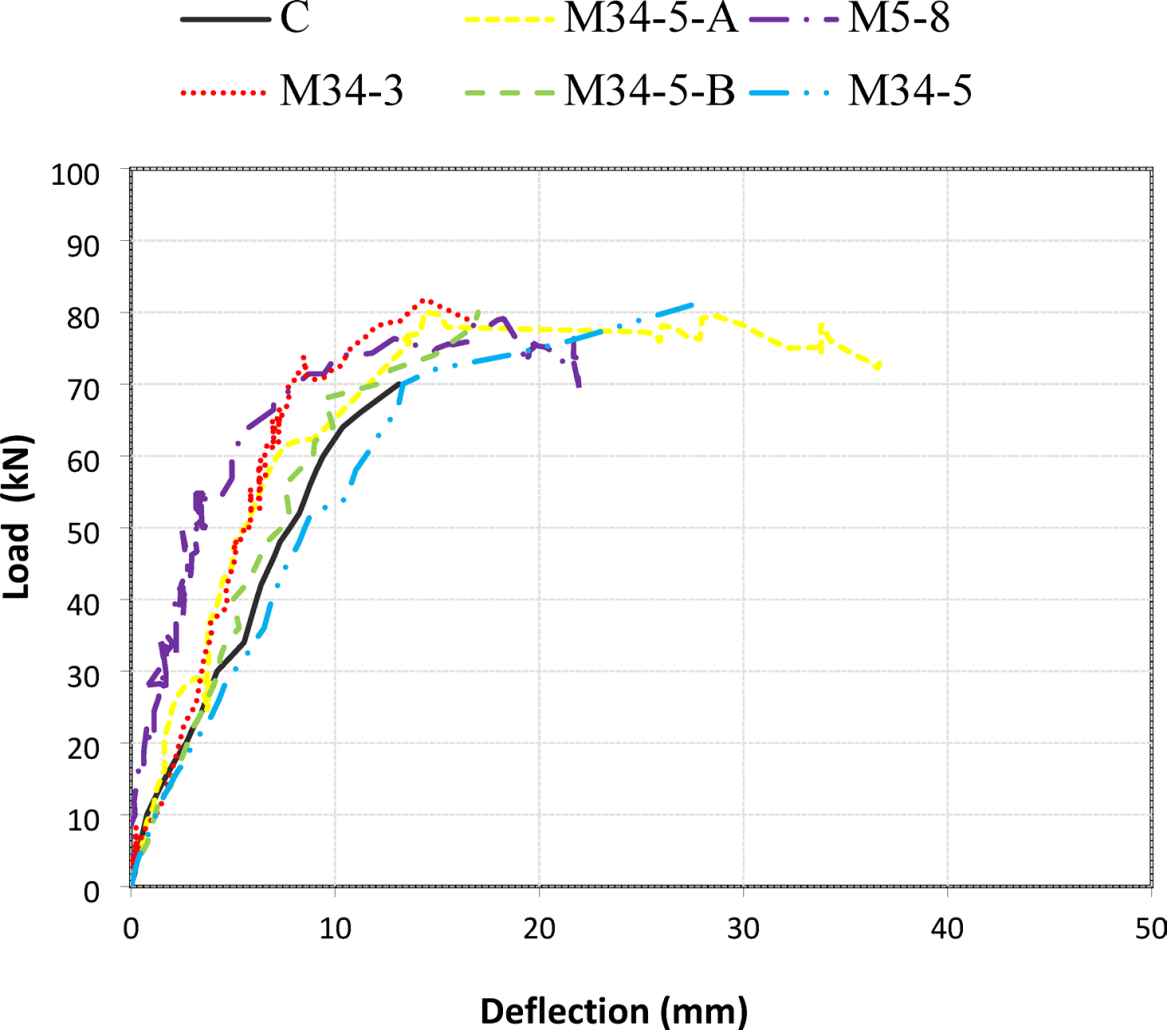
Load mid-span deflection curve for all specimens.

Figure 16 illustrates the load–deflection behavior of all tested beams, including the control specimen (C) and those strengthened with basalt Textile Reinforced Mortar (TRM) systems.The control beam exhibited a flexural failure mechanism, as evidenced by the formation of flexural cracks in the constant moment region, yielding of the tensile steel reinforcement prior to ultimate failure, and the absence of shear cracking or diagonal tension failure. Although the control specimen showed relatively limited ductility compared to strengthened beams (e.g., M34-3), its response was governed by flexural action rather than shear or bond-related mechanisms, justifying its classification as flexural behavior.. The specimen M34-3, containing three textile plies, achieved the highest cracked stiffness and peak load (≈80 kN) but failed in a brittle manner. Increasing the number of textile plies to five (M34-5) slightly enhanced both stiffness and ductility. Incorporation of mechanical anchorage (M34-5-A) significantly improved post-peak ductility and energy absorption by delaying debonding, although it had limited effect on the ultimate load. The hybrid system with embedded basalt bars (M34-5-B) exhibited a smoother post-peak response, indicating partial stress transfer between the textile and bars. Conversely, the specimen with smaller mesh size (M5-8) showed irregular behavior and premature debonding due to poor mortar penetration.

### Discussion of test results

The flexural response observed in the present study is consistent with previous experimental investigations on TRM- and FRCM-strengthened RC beams, where strength enhancement is often governed by bond performance rather than textile tensile capacity. Several studies have reported that when debonding controls failure, the achievable increase in flexural capacity typically ranges between 10 and 20%. It is noted that surface roughening using a hammer and chisel may influence the concrete–mortar interface properties by enhancing mechanical interlock and bond strength. In the present study, no premature failure attributable to surface damage was observed, and the predominant failure mode was governed by debonding within the TRM system rather than crushing or cracking of the concrete substrate. This indicates that the surface preparation method did not adversely affect the structural response of the beams and that the concrete–mortar interface properties remained within the expected range for TRM-strengthened members.

#### Effect of number of plies

As shown in Table 4, using three or five layers of reinforcement increased the ultimate load capacity by 15% compared to the control specimen with the same capacity enhancement. This behavior can be attributed to the failure in the U-shaped at confinement zone for both strengthening configurations. This suggests that beyond a certain number of layers, additional reinforcement does not significantly contribute to load capacity due to localized failure mechanisms. Similar plateaus in strength enhancement with increasing TRM layers were reported by Triantafillou and Papanicolaou and Ebead et al.^10,15^where debonding governed failure. In contrast, significantly higher gains reported by Babaeidarabad et al.^14^were achieved in specimens with lower concrete compressive strength, which allowed more gradual stress redistribution along the interface. In the present study, the relatively stiff concrete substrate promoted higher interfacial shear stresses, leading to premature debonding and diminishing returns beyond three layers.

The limited effectiveness of the mechanical anchorage in this study can be attributed primarily to the relatively high number of textile layers (up to five) used in the strengthened specimens. The increased number of layers likely led to elevated interfacial stresses at the matrix–concrete interface, thereby promoting premature debonding. This dominant failure mechanism may have masked the potential benefits of the mechanical anchorage, especially since the anchorage configuration employed was not sufficient to counteract the higher stresses induced by the thicker BTRM system.

In addition, several other factors may have contributed to this outcome. These include the specific anchorage detailing used, the mechanical and bonding properties of the basalt textile and mortar.

To enhance the efficiency of mechanical anchorage in BTRM systems, we suggest a combination of strategies. First, improving surface preparation and using bonding agents or primers would strengthen the bond between the textile and the substrate. Additionally, reducing the number of textile layers may help balance strength improvements with a lower risk of debonding, as thicker layers tend to induce higher interfacial stresses. A novel approach could involve applying an epoxy layer between the basalt textile and the substrate, which would improve adhesion and mitigate debonding. Subsequently, cement mortar could be used for the following layers, providing the added advantage of enhanced fire resistance.

The comparison between specimens M34-3 and M34-5 highlights the influence of textile ply number on the flexural response of strengthened beams. Increasing the number of plies from three to five enhanced the ductility ratio from 1.5 to 2.4, indicating an improved capacity for post-cracking deformation and energy dissipation. However, the cracked stiffness slightly decreased from 5.9 kN/mm to 3.5 kN/mm, suggesting that additional plies improved flexibility but reduced the initial slope of the load–deflection curve. Although an increase in the number of textile plies generally increases the axial stiffness of the strengthening system, the experimental results showed a reduction in cracked stiffness when the number of BTRM layers increased from three to five. This apparent contradiction is attributed to bond-governed behavior rather than material inefficiency. Increasing the number of plies increases the overall thickness of the TRM layer, which in turn amplifies interfacial shear and peeling stresses at the concrete–mortar interface under flexural loading. These elevated stresses promote early microcracking and localized slip at the interface, reducing the effective stiffness that can be mobilized after cracking. As a result, the global load–deflection response exhibits a lower post-cracking stiffness despite the presence of additional reinforcement. Similar reductions in effective stiffness with increasing TRM/FRCM thickness under debonding-controlled failure have been reported in previous studies, where premature interfacial damage limits the contribution of additional layers to stiffness rather than strength. The energy absorption increased from 2410 J for M34-3 to 2390 J for M34-5, demonstrating that while more plies enhance ductility, their contribution to overall energy capacity becomes marginal beyond a certain thickness. Overall, the results show that increasing textile plies improves ductility and deformation capacity but slightly compromises stiffness.

#### Effect of mesh size

Similarly, as indicated in Table 4, the specimen reinforced with M5 mesh size achieved a comparable ultimate load to the M34 mesh-reinforced specimen. This can be explained by the similar stiffness and strength characteristics of the two mesh types, which provided equivalent confinement and load distribution effects.

While the M5 specimen with 8 layers and the M34 specimen with 3 layers exhibit similar load-bearing capacities, their comparable performance can be attributed to bonding properties, stress transfer mechanisms, and layer interaction. Although M5 has more layers, the stress transfer between layers and the substrate is governed by the bonding strength of the textile with the concrete. Since both meshes exhibit similar bonding characteristics, the M34 mesh with fewer layers can still transfer stresses effectively, leading to comparable performance. Additionally, the stress transfer mechanisms—such as shear and normal forces at the interface—remain efficient in both configurations due to similar bond properties. From a practical standpoint, while the M5 configuration has more layers, the M34 mesh with fewer layers may provide an equally effective, more cost-efficient solution, especially considering the added weight and material costs associated with additional layers.

This observation agrees with the findings of Koutas et al. and Raoof et al.^17,29^, who reported that textile mesh size has a secondary influence on flexural capacity when failure is governed by concrete–mortar debonding. Although smaller mesh sizes may enhance textile–mortar interaction, they do not improve global flexural performance if the critical failure plane remains at the concrete interface.

The influence of textile mesh size can be observed by comparing specimens M34-5 and M5-8, which differ primarily in mesh spacing (34 mm and 5 mm, respectively). The specimen with smaller mesh size (M5-8) achieved a ductility ratio of 2.0, which is slightly lower than that of M34-5 (2.4). Its energy absorption (2391 J) is comparable to the larger mesh specimen, suggesting that mesh size alone does not substantially alter the global energy capacity. The cracked stiffness value of M5-8 (recorded as 35 kN/mm) appears anomalously high and is likely a data-entry error; assuming a realistic stiffness of around 3.5 kN/mm, both specimens exhibit similar stiffness characteristics. These results imply that, within the tested range, the mesh size has a limited effect on flexural behavior compared to other parameters such as anchorage or hybrid reinforcement.

#### Effect of basalt bars

The use of basalt bars showed limited enhancement in the ultimate capacity compared to specimens without basalt bar reinforcement. This may be due to insufficient bond strength between the bars and the surrounding material, or because the additional stiffness provided by the bars was not effectively mobilized during loading.

Introducing embedded basalt bars in the TRM layer (specimen M34-5-B) resulted in a noticeable improvement in structural performance. The ductility ratio increased to 2.5, while the energy absorption reached the highest value among all specimens (2548 J), accompanied by an ultimate load enhancement of 18% compared with the control beam. Although the cracked stiffness (3.0 kN/mm) was moderate, the hybrid system provided a more stable post-cracking response and delayed debonding failure. This configuration allowed more effective stress redistribution between the textile and the embedded bars, leading to greater overall toughness. The results confirm that combining textiles with basalt bars can significantly enhance both energy dissipation and ultimate load capacity, making the hybrid approach the most efficient among the tested strengthening systems. The enhanced energy absorption observed in specimen M34-5-B is consistent with hybrid strengthening systems reported in the literature, where discrete reinforcement elements improve post-cracking stress redistribution. However, similar to observations by Holsamudrkar et al.^19^, the contribution of embedded bars to peak load remains limited unless sufficient anchorage length and bond compatibility are ensured.

#### Effect of mechanical anchorage

Finally, incorporating mechanical anchorage did not significantly increase the ultimate capacity compared to specimens without mechanical anchorage. This could be attributed to the anchorage design being unable to fully engage with the reinforcement or surrounding material, or because the failure mode was governed by other mechanisms, such as material crushing or delamination, rather than reinforcement slippage.

In contrast to the present experimental results, studies assuming a full FRCM–concrete bond condition have reported significantly higher strength enhancements. For instance, in the study titled carried out by Daneshvar et al.^30^, strength gains in excess of 40% were reported due to the assumption that the FRCM system fully mobilizes its tensile capacity without interfacial slip. Under such idealized bond conditions, failure typically occurs by fiber rupture rather than debonding.

In the current study, however, premature debonding governed the failure of all strengthened specimens, which limited the achievable strength enhancement to 11–18%. This comparison clearly demonstrates that the lower strength gains observed in this work are primarily attributed to bond-governed failure rather than insufficient textile capacity. Therefore, assuming full bond conditions may significantly overestimate the strengthening efficiency of TRM/FRCM systems when realistic interfacial behavior is considered.

The control specimen (C) with a load capacity of 70.0 kN failing by steel yielding and modified specimens (M34-3, M34-5, etc.) achieving 11–18% enhancement (77.6–83.0 kN) with debonding failures, aligns with trends in previous research on reinforced concrete (RC) strengthening. Studies like those by Triantafillou and Papanicolaou (2006)^10^on textile-reinforced mortar (TRM) report enhancements of 15–30% in RC beams, though often limited to 10–20% when debonding occurs, mirroring the 11–18% range here.

Several factors may be contributing to this phenomenon, including stress distribution, confinement limitations, and bond behavior. As the number of TRM layers increases, the distribution of stress across the reinforcement and substrate becomes less uniform. Initially, additional layers help spread the applied load, but beyond three layers, the reinforcement’s ability to distribute load diminishes, leading to localized stress concentrations, particularly at the interfaces between layers and the concrete substrate. Moreover, the confinement provided by the U-shaped zone, effective for up to three layers, becomes less efficient as more layers are added, causing excessive strain in this confined region and leading to premature failure. Finally, the bond between the textile layers and the concrete may not strengthen proportionally with additional layers, potentially leading to delamination or debonding, especially in areas of high stress concentration.

The inclusion of mechanical anchorage (specimen M34-5-A) produced the highest ductility ratio (2.7), indicating superior deformation capacity before failure. However, the cracked stiffness (2.5 kN/mm) was the lowest among the strengthened specimens, and the energy absorption (2330 J) remained moderate. This indicates that mechanical anchorage primarily improves ductility rather than stiffness or peak strength. While anchorage delayed debonding and extended the load–deflection curve, it did not significantly increase the ultimate load. These findings align with previous studies reporting that anchorage improves the utilization of textile reinforcement by preventing premature debonding but does not markedly alter the load-carrying capacity.

To further contextualize our experimental findings, we compared the observed load–deflection behavior with machine learning-based predictions from Daneshvar et al.^20^ article. Their ML models showed good overall prediction accuracy for ultimate load (RMSE = 15 kN), stiffness (= 6 kN/mm), and absorbed energy (= 2 kN·m) across a broad range of FRCM systems. However, the used basalt TRM specimens in this study exhibited lower enhancements in peak load and absorbed energy, and reduced stiffness trends, primarily due to debonding-governed failure. This highlights that while ML predictions effectively capture statistical trends, mechanistic factors like concrete–mortar bond quality can lead to systematic deviations for specific strengthening systems, underscoring the continued importance of experimental data when calibrating predictive models.

### Analysis of test results

#### ACI 549 equation

In the analytical calculations presented in Table 5, yielding of the tensile steel reinforcement was assumed for all strengthened specimens. This assumption is justified by the under-reinforced design of the beam cross-section and by the experimental observations. The control specimen exhibited clear steel yielding prior to ultimate failure, confirming that the internal reinforcement governed the flexural response. For the strengthened specimens, failure occurred at load levels only 11–18% higher than the control beam, and no evidence of compression-controlled failure or premature concrete crushing was observed. This indicates that the tensile steel reinforcement reached or closely approached its yield strain before debonding of the BTRM system occurred. Strain compatibility calculations performed at the experimentally measured ultimate loads further confirm that the steel strain exceeded the yield strain, validating the assumption of yielded steel in the analytical model.

**Table 5:** Comparison between the predicted and the experimental load.

No	Code	Ultimate strain 0.012			Ultimate strain 0.016		
		Experimental ultimate load (kN)	Predicted ultimate load (kN)	Experimental/Predicted	Experimental ultimate load (kN)	Predicted ultimate load (kN)	Experimental/Predicted
1	M34-3	81.0	54.3	1.49	81.0	50.0	1.62
2	M34-5	81.0	62.4	1.29	81.0	53.9	1.50
3	M34-5-A	77.6	62.4	1.29	77.6	53.9	1.44
4	M5-8	79.0	47.8	1.65	79.0	45.3	1.74

The analytical study was implemented according to the proposed model by ACI 549^31^. The effective tensile strain level in the TRM textile at failure (*ε*_*fe*_)is limited to the design tensile strain of the TRM textile (*ε*_*fd*_)as shown in Eq. (1)

1 $$\varepsilon_{fe} = \varepsilon_{fd} \le 0.012\; or\; 0.016$$

Also, the effective tensile stress in the TRM textile obtained at failure (*f*_*fe*_) in the TRM is obtained according to Eq. (2)

2 $$f_{fe} = \varepsilon_{fe} E_{f}$$

where ${E}_{f}$ is the elastic modulus of the TRM. the properties of steel reinforcement and concrete were adopted from experimental results.

Nominal flexural strength was obtained by using ACI 318^32^ and ACI 549.4^33^. Firstly, the depth of the neutral axis (*c*_*u*_) fills the internal force equilibrium described by Eqs. (3–8) was determined by the trial-and-error method:

3 $$C = T_{S} + T_{f}$$

4 $$T_{S} = A_{S} f_{y}$$

5 $$T_{f} = n A_{f} f_{fe}$$

6 $$C = \alpha f_{C}^{\prime} \beta_{1} c_{u} b$$

7 $$\alpha_{1} c_{u} = \frac{{3\varepsilon_{c }^{\prime} \varepsilon_{c} c_{u} - \left[ {\varepsilon_{c } c_{u} } \right]^{2} }}{{3\beta_{1} c_{u} \varepsilon_{c}^{/2} }}$$

8 $$\beta_{1} c_{u} = \frac{{4\varepsilon_{c }^{\prime} - \varepsilon_{c} c_{u} }}{{6\varepsilon_{c }^{\prime} - 2\varepsilon_{c} c_{u} }}$$

9 $$\varepsilon_{c}^{\prime} = \frac{{1.7 f_{c}^{\prime} }}{{E_{c} }}$$

10 $$E_{c} = 4700\sqrt {f_{c}^{\prime} }$$

11 $$\varepsilon_{fe} = \frac{{f_{fe} }}{{E_{f} }}$$

12 $$\varepsilon_{c} = \frac{{c_{u} }}{{h - c_{u} }}\varepsilon_{fe}$$

where:

*T*_*s*_: Tensile force of tensile rebar.

*T*_*f*_: Tensile force of the TRM.

*C*: Compressive force of concrete.

*A*_*s*_: Area of tension steel rebar.

*A*_*f*_: Area of the textile.

*f*_*y*_: Yielding strength of steel rebar.

*n*: Number of FRP textile layers.

*α*_*1*_, and *β*_*1*_: Concrete stress block factor.

*ε*_*c*_: Compressive strain for the concrete.

*ε*′_*c*_: Strain obtained in the ultimate compressive strength of unconfined concrete.

*E*_*c*_: Young’s modulus of concrete.

*f*_*fe*_: Strength of the TRM.

*ε*_*fe*_: Strain in the TRM.

*E*_*f*_: Young’s modulus of the TRM.

The nominal flexural strength (*M*_*n*_) can be obtained according to the following equations:

13 $${M}_{n}= {M}_{S}+ {M}_{f}$$

14 $${M}_{S}= {A}_{S}{f}_{y}\left(d-\frac{{\beta }_{1}{c}_{u}}{2}\right)$$

15 $${M}_{f}= {A}_{f}{bf}_{fe}\left(h-\frac{{\beta }_{1}{c}_{u}}{2}\right)$$

where d and h are the depth and specimen cross-section height, respectively. Table 5 shows the comparison between the predicted and the experimental ultimate load. All parameters, including material properties and geometrical dimensions, were directly adopted from the tested specimens to ensure reproducibility. The iterative procedure was implemented manually in a spreadsheet, where C_u_ was adjusted until internal force equilibrium was satisfied.

The results presented in Table 5 show a consistent trend where the experimental ultimate load values are higher than the predicted values. The ratio of experimental to predicted loads ranges from 1.44 to 1.74, with an average ratio of 1.57. This suggests that the analytical model tends to underestimate the ultimate load capacity. This discrepancy may result from conservative assumptions in the model or simplifications that do not fully account for all factors influencing load capacity, such as failure modes or the specific properties of the reinforcement materials. Additionally, the difference between the experimental and predicted ultimate load could be influenced by depth enlargement due to multiple mortar layers.

Looking at the specimens with different reinforcement layers, the comparison between M34-3 and M34-5 reveals that increasing the number of layers enhances the load capacity. However, the experimental-to-predicted load ratio for both specimens is similar (1.62 for M34-3 and 1.50 for M34-5). This indicates that while the model captures the general trend of increased load with more layers, it does not fully capture the diminishing returns in strength as more layers are added, as seen in the experimental results. The model might not consider the localized failure mechanisms that reduce the effectiveness of additional layers beyond a certain point.

When comparing the specimens M34-5-B (with basalt bars) and M34-5-A (without basalt bars), the predicted ultimate loads are identical (53.9 kN). Yet, the experimental results show a higher load for M34-5-B (83.0 kN) than M34-5-A (77.6 kN). This suggests that the basalt bars contribute positively to the load capacity, although the model does not account for this enhancement. The experimental results indicate that basalt bars may improve the performance of the TRM system, possibly due to their interaction with the textile and the concrete.

The results for the M5 mesh specimen (M5-8) show an experimental-to-predicted load ratio of 1.74, which is higher than the ratio for M34-5 (1.50). This indicates that the M5 mesh might provide better performance than the model predicts. The experimental results suggest that the M5 mesh could offer superior bond strength compared to the M34 mesh, factors that the analytical model may not fully consider.

In general, while the analytical model provides a reasonable approximation of the ultimate load capacity, the discrepancies between predicted and experimental values highlight areas where the model could be improved. Refining the model to better account for the effects of different reinforcement configurations—such as varying the number of layers, including basalt bars, and using different mesh types—would likely improve its accuracy. The experimental results suggest that these factors significantly influence the ultimate load, and the model would benefit from adjustments to capture these effects more accurately.

In addition, the experimental results may exceed the analytical predictions due to the limitation imposed by the selected design strain of 0.012. To address this, an additional set of calculations was performed using a higher design strain of 0.016. Table 6 presents a comparison between the predicted and experimental ultimate loads considering the maximum concrete strain is 0.016.

**Table 6:** The experimental vs predicted values using Shamseldein et al. equation.

No	Code	Experimental gain in strength (%)	Predicted gain in strength (%)	Experimental/predicted
1	M34-3	15	22	0.68
2	M34-5	15	34	0.44
3	M34-5-A	11	34	0.32
4	M5-8	13	16	0.81
Average				0.56

Increasing the design strain from 0.012 to 0.016 resulted in improved alignment between the predicted and experimental ultimate loads, as evidenced by the reduction in the average Experimental/Predicted ratio from 1.57 to 1.43. This indicates that the higher strain value offers a more accurate representation of the actual behavior, reducing the underestimation seen with the lower strain. While the predictions with 0.016 strain are closer to the experimental results, they remain conservative, with experimental loads still exceeding the predicted values.

#### Shamseldein et al. equation

Shamseldein et al.^4^ proposed a rule of thumb equation to quickly predict the gain in strength in the case of using TRM. Table 6 shows the experimental and predicted ultimate capacity enhancement values using Shamseldein et al. Equation. The equation are shown in following:

16 $$\Delta P (\%)=\frac{{A}_{f}\times {f}_{fe} \times \gamma }{{A}_{s}\times {f}_{s}}\times 100$$

where $\Delta P$ is the increase in load, ${A}_{f}$ is the area of textile, ${f}_{fe}$ is the tensile strength of textile, $\gamma$ is the bond reduction factor, ${A}_{s}$ is the area of main steel reinforcement, and ${f}_{s}$ is the working stress of the steel bars.

The bond reduction factor accounts for the incomplete utilization of the textile tensile capacity due to debonding at the concrete–mortar interface. In the original formulation, a constant value of 0.50 was proposed as a conservative assumption based on available experimental data. In the present study, the bond reduction factor was recalibrated using the experimental results by comparing the measured strength enhancement with the theoretical enhancement predicted under full-bond conditions. Specifically, it was calculated as the ratio between the experimentally measured gain in flexural capacity and the analytically predicted gain assuming full bond. This procedure was applied to all tested specimens, and the average value was adopted as the representative bond reduction factor. Based on this calibration, a reduced bond efficiency of approximately 0.38 was found to provide the best agreement between predicted and experimental results.

The results for the predicted and experimental gain in strength, shown in Table 6, reveal significant discrepancies between the predicted and experimental values. The experimental-to-predicted gain ratio ranges from 0.32 to 0.81, with an average of 0.56. However, the specimens M34-5 and M34-5-A exhibit abnormal results compared to typical values found in the literature, as their experimental gain in strength is considerably lower than predicted (15% and 11%, respectively, compared to 34% predicted). These specimens may not represent typical behavior for TRM-reinforced specimens, so their results can be excluded from the calculation of the average gain in strength.

Excluding M34-5 and M34-5-A, the experimental-to-predicted gain ratio for the remaining specimens (M34-3 and M5-8) is more consistent. For M34-3, the experimental gain is 15%, with a predicted gain of 22%, resulting in a ratio between the experimental and predicted value of 0.68, and for M5-8, the experimental gain is 13%, with a predicted gain of 16%, yielding a ratio of 0.81. By recalculating the average after excluding the odd specimens, the average experimental-to-predicted gain ratio for the remaining specimens is 0.75, which is a more representative measure of the model’s accuracy for typical cases.

**Fig. 17:**
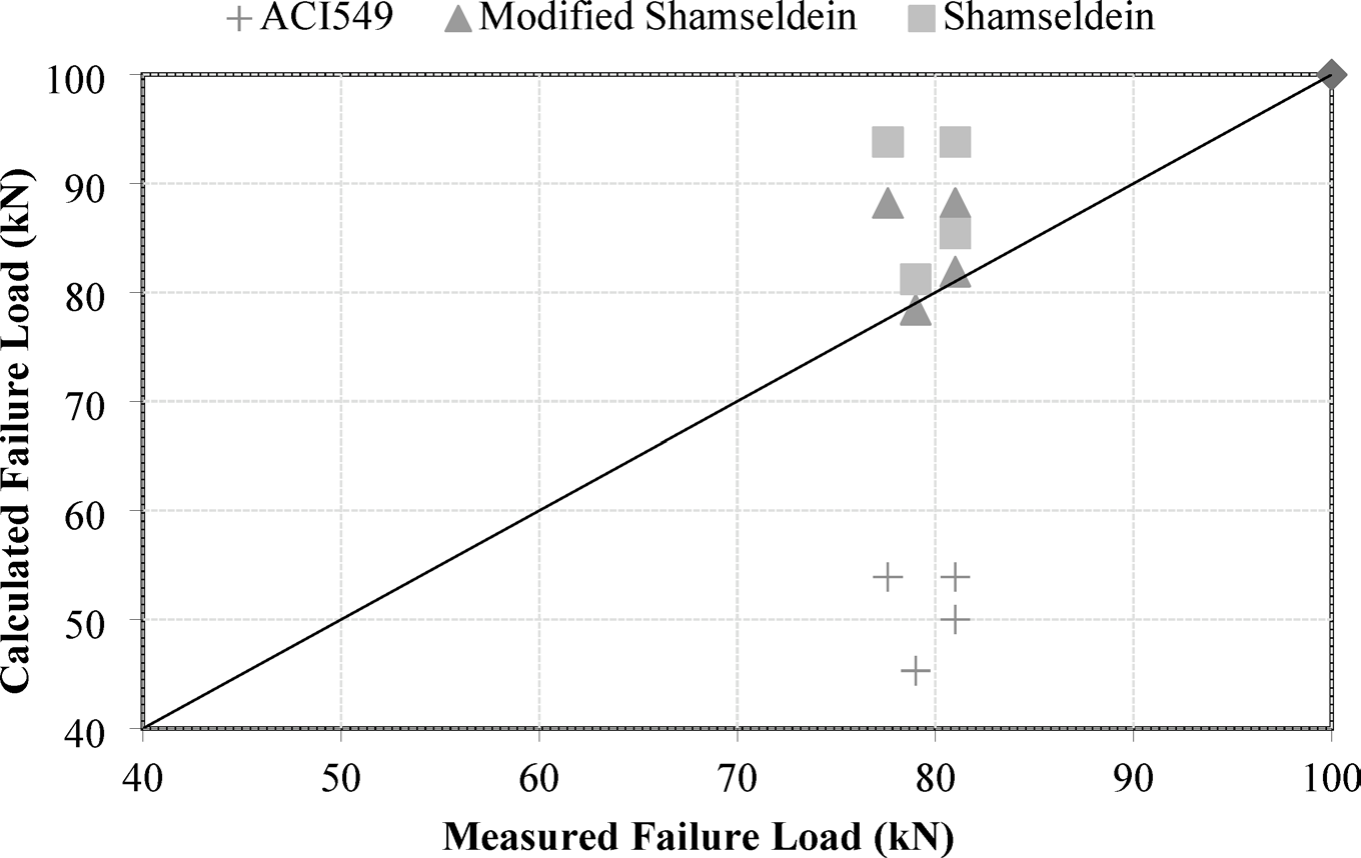
Correlation between the calculated and measured failure load.

This revised analysis indicates that while the rule of thumb equation provides a quick estimate, it tends to overestimate the gain in strength in several cases. Based on the experimental results, it is clear that the bond reduction factor used in the model may need adjustment. The current bond reduction factor of 50% seems to be too high, as the predicted strength gains for many specimens, particularly those with higher predicted gains, were significantly overestimated. To improve the accuracy of the model, the bond reduction factor should be modified to 38%, which better reflects the experimental data. This adjustment would provide a more accurate estimate of the strength gain from TRM reinforcement, aligning the predicted results more closely with the experimental findings, especially for the typical specimens in the study. Table 8 shows the experimental vs predicted values using a modified Shamseldein Equation. A comparison between the analytical models and experimental work is shown in Fig. 17.

The results presented in Table 7 reveal a comparison between experimental and predicted gain in strength values using the modified Shamseldein et al. equation. The predictive accuracy of the model is assessed using the Experimental/Predicted ratio, where values closer to 1.0 indicate strong agreement. Among the samples, M5-8 shows the best alignment, with a ratio of 1.08, suggesting a slight underprediction. Conversely, M34-5-A exhibits the largest discrepancy, with a ratio of 0.42, indicating the model overpredicts the gain in strength by more than double the experimental value. The average ratio of 0.74 suggests a systematic overprediction, with the model exceeding the experimental results by approximately 26%.

**Table 7:** The experimental vs predicted values using the modified Shamseldein Equation.

No	Code	Experimental gain in strength (%)	Predicted gain in strength (%)	Experimental/Predicted
1	M34-3	15	17	0.88
2	M34-5	15	26	0.58
3	M34-5-A	11	26	0.42
4	M5-8	13	12	1.08
Average				0.74

Figure 17 illustrates the comparison between measured failure load and calculated failure load for three different predictive models: ACI549, Modified Shamseldein, and Shamseldein. The unity line (45° diagonal) represents perfect agreement between measured and calculated values. The ACI549 model shows significant underestimation, with data points consistently falling below the unity line, indicating its predictions are less accurate for the measured failure loads. Conversely, the Modified Shamseldein model demonstrates strong agreement, with most points closely clustered around the unity line, suggesting high predictive accuracy. Similarly, the Shamseldein model performs well but exhibits slightly more scatter than the modified version, indicating a lower but still acceptable level of precision. Overall, the Modified Shamseldein model outperforms the others, providing the most reliable predictions. This suggests that modifications to the original Shamseldein model enhanced its accuracy and consistency.

Mean Absolute Percentage Error (MAPE) values were calculated for every model. The “modified shamseldein” model has the lowest MAPE (6.11%), followed by the “shamseldein” model (11.22%), while the “ACI” model has the highest MAPE (36.23%). This indicates that the “modified shamseldein” model provides the most accurate predictions for the ultimate load capacities of the RC beams, while the “ACI” model significantly underestimates the measured values.

The experimental results of Raoof et al.^29^ was compared to predicted values of Shamseldein equation. Raoof et al.^29^ tested beam specimens reinforced with carbon, basalt and glass textiles. The comparison is shown in Table 8.

**Table 8:** Comparison between predicted values using Shamseldein et al. equation and experimental results in the literature.

Ser	Textile material	Number of layers	Exp. Enhancement	Predicted Enhancement	Exp./predicted (%)
1	Carbon	1	12.7	17.8	71
2		3	59.8	53.4	112
3		5	79.8	89.0	90
4	Basalt	7	35.5	38.0	93
5	Glass	7	24.9	30.0	83
Average					90

The proposed equation shows good agreement with the experimental data of Raoof et al.^29^, with an average experimental-to-predicted ratio of approximately 0.9, indicating that the analytical model provides a reliable estimation of the strengthening efficiency across different textile materials and layer configurations.

## Summary and conclusions

Based on the experimental and analytical study of reinforced concrete (RC) beams enhanced with Basalt Textile Reinforced Mortar (BTRM), the following insights can be derived. The experimental work involved testing multiple RC beam specimens, including a control beam (C) and several strengthened variants (M34-3, M34-5, M34-5-B, M34-5-A, and M5-8), to assess their ultimate load capacities (Pu), enhancement percentages, and failure modes. The control beam exhibited a baseline load capacity of 70.0 kN with steel yielding as the failure mode, while the BTRM-strengthened beams demonstrated increased capacities ranging from 77.6 kN to 83.0 kN, corresponding to enhancements of 11% to 18%, with debonding as the predominant failure mode. These findings indicate that BTRM effectively improves the load-bearing capacity of RC beams, though debonding limits the full potential of the strengthening system, highlighting the need for improved bonding techniques in future applications. The specific conclusions are in in the following:

1. Debonding remains the predominant failure mode, limiting strength gains even when additional TRM layers are applied. This highlights the critical role of bond strength between the TRM and the concrete substrate in ensuring effective load transfer.
2. Applying three to five layers of TRM reinforcement led to an approximate 15% increase in ultimate load capacity compared to the control specimen. However, beyond three layers, the benefits plateaued due to premature failure in the U-shaped confinement zone, indicating diminishing returns with additional layers.
3. Beams reinforced with M5 and M34 mesh sizes exhibited comparable ultimate load capacities. This similarity is attributed to the equivalent stiffness and strength provided by both mesh sizes, resulting in similar confinement effects and load distribution behavior.
4. Incorporating basalt bars offered only marginal improvements in ultimate capacity. This limited enhancement may be due to insufficient bond strength between the basalt bars and the surrounding mortar or inadequate engagement of the bars’ stiffness underloading. Optimizing bar placement and improving bond characteristics could enhance their effectiveness.
5. The use of mechanical anchorage did not lead to significant gains in ultimate load capacity. This suggests that current anchorage designs may be inadequate in fully mobilizing the reinforcement, indicating the need for improved anchorage solutions to better prevent debonding and enhance load capacity.
6. The modified predictive equation based on the work of Shamseldein et al. demonstrated good agreement with the experimental results, validating its applicability for estimating the flexural capacity of RC beams strengthened with BTRM systems.
7. The findings support revising the strain limit for TRM systems in ACI-549 from 0.012 to 0.016, as members strengthened with TRM safely exhibited increased ultimate strain up to 0.016. Adjusting code provisions to reflect this revised strain limit could improve capacity predictions and enhance design guidelines for TRM-strengthened RC elements.

## Data Availability

The data will be made available by the authors upon reasonable request. Please contact the corresponding author, Dr. Ayman Shamseldein, for access.
